# Supplementary material for: Identification of Candidate Susceptibility Genes to Puccinia graminis f. sp. tritici in Wheat
Source: Front Plant Sci. 2021 Apr 21;12:657796. doi: 10.3389/fpls.2021.657796 (PMC8097158; doi:10.3389/fpls.2021.657796)
Supplement: Supplementary file 8 [file Data_Sheet_1.pdf]

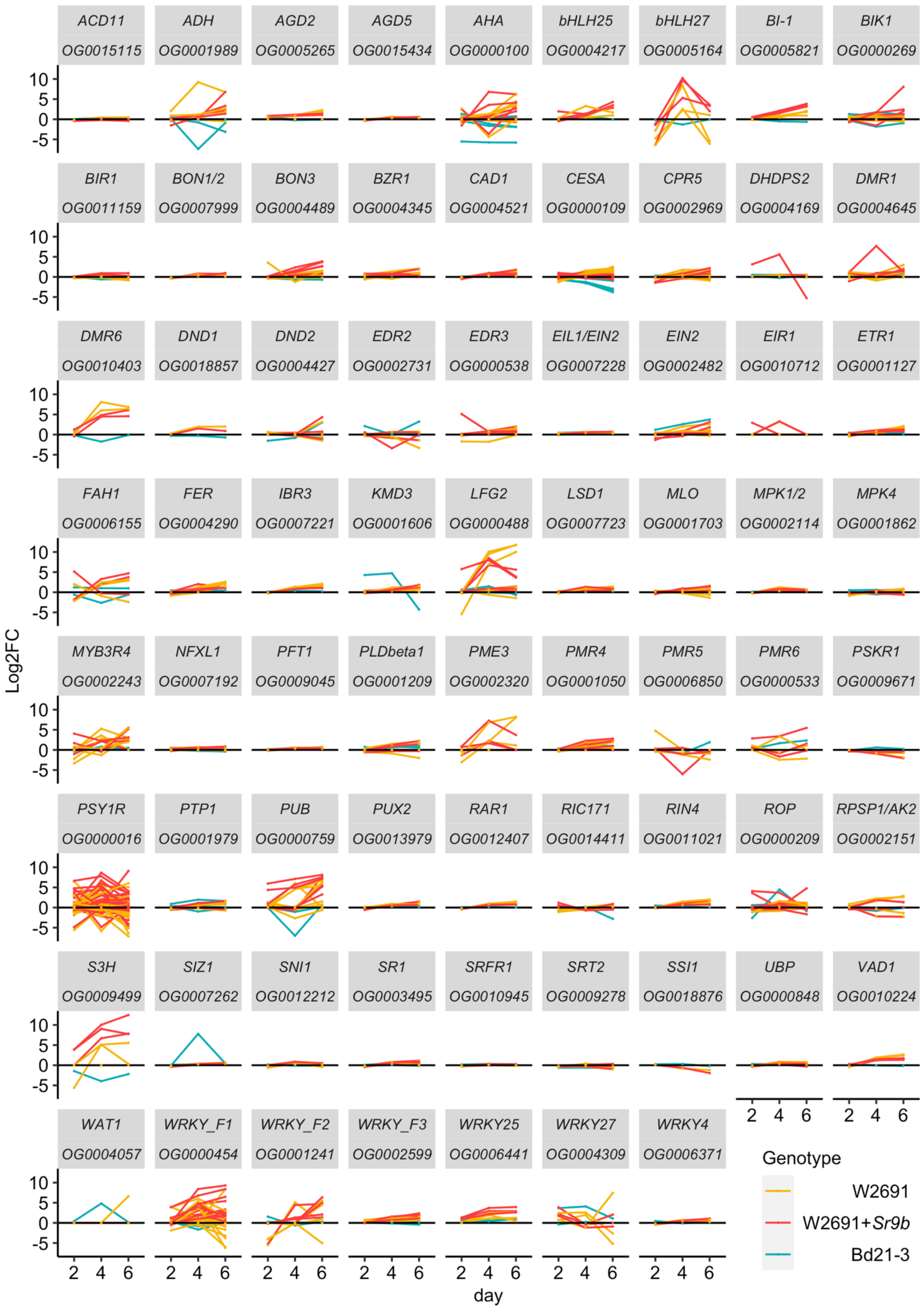
ACD11 OG0015115
ADH OG0001989
AGD2 OG0005265
AGD5 OG0015434
AHA OG0000100
bHLH25 OG0004217
bHLH27 OG0005164
BI-1 OG0005821
BIK1 OG0000269
BIR1 OG0011159
BON1/2 OG0007999
BON3 OG0004489
BZR1 OG0004345
CAD1 OG0004521
CESA OG0000109
CPR5 OG0002969
DHDPS2 OG0004169
DMR1 OG0004645
DMR6 OG0010403
DND1 OG0018857
DND2 OG0004427
EDR2 OG0002731
EDR3 OG0000538
EIL1/EIN2 OG0007228
EIN2 OG0002482
EIR1 OG0010712
ETR1 OG0001127
FAH1 OG0006155
FER OG0004290
IBR3 OG0007221
KMD3 OG0001606
LFG2 OG0000488
LSD1 OG0007723
MLO OG0001703
MPK1/2 OG0002114
MPK4 OG0001862
MYB3R4 OG0002243
NFXL1 OG0007192
PFT1 OG0009045
PLDbeta1 OG0001209
PME3 OG0002320
PMR4 OG0001050
PMR5 OG0006850
PMR6 OG0000533
PSKR1 OG0009671
PSY1R OG0000016
PTP1 OG0001979
PUB OG0000759
PUX2 OG0013979
RAR1 OG0012407
RIC171 OG0014411
RIN4 OG0011021
ROP OG0000209
RPSP1/AK2 OG0002151
S3H OG0009499
SIZ1 OG0007262
SNI1 OG0012212
SR1 OG0003495
SRFR1 OG0010945
SRT2 OG0009278
SSI1 OG0018876
UBP OG0000848
VAD1 OG0010224
WAT1 OG0004057
WRKY_F1 OG0000454
WRKY_F2 OG0001241
WRKY_F3 OG0002599
WRKY25 OG0006441
WRKY27 OG0004309
WRKY4 OG0006371
Log2FC
10
5
0
-5
2 4 6
day
Genotype
W2691
W2691+Sr9b
Bd21-3

Supplementary Figure 1. Expression profile patterns of orthogroups containing candidate *S* genes in *T. aestivum* (W2691 and W2691+*Sr9b*) and *B. distachyon* (Bd21-3) genotypes throughout infection with *P. graminis* f. sp. *tritici*. Log2 fold change values (y-axis) for all gene orthologs are presented per sampling time point (x-axis). Name of *S* gene and orthogroup identifier are shown in each graph.

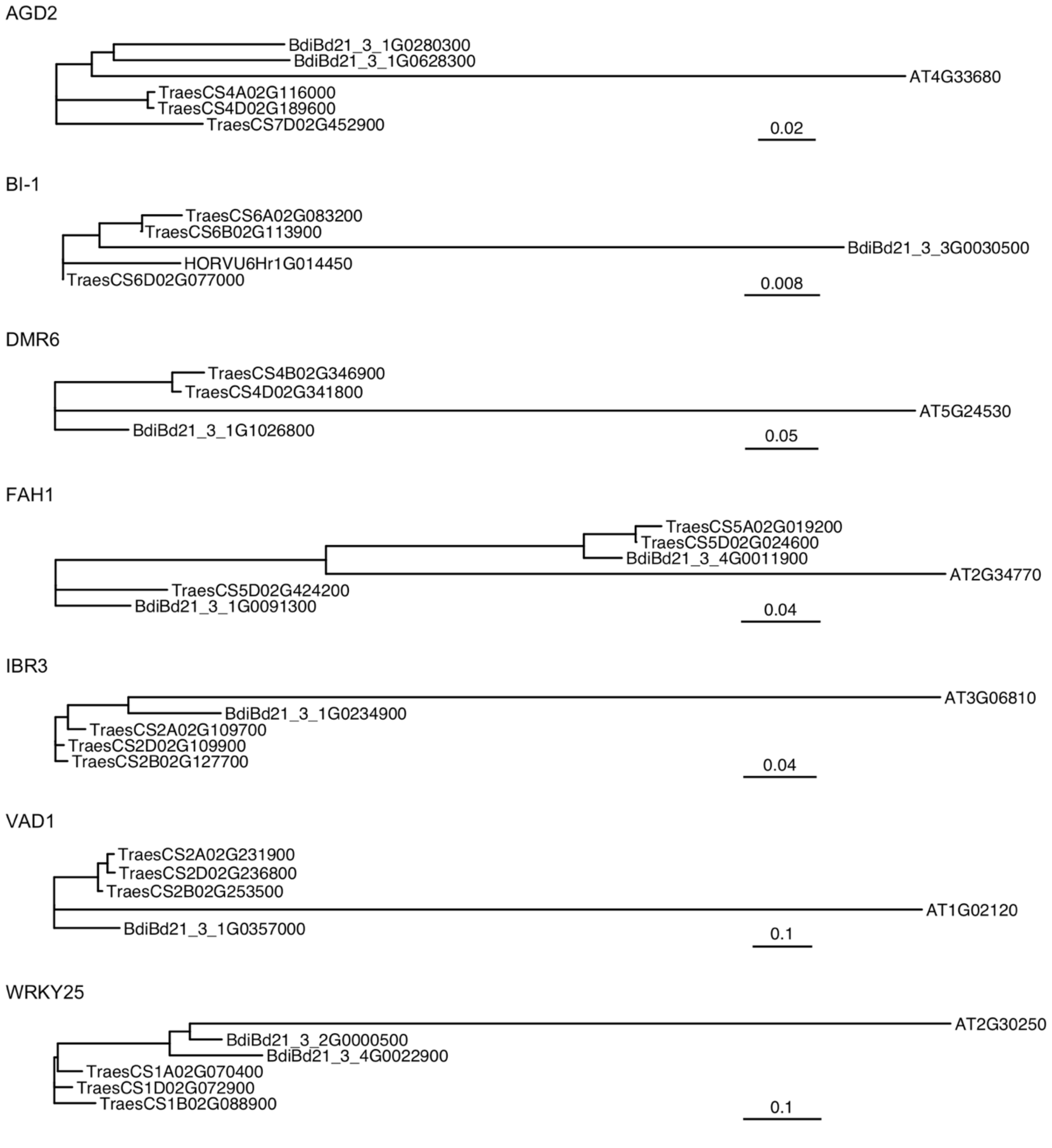

Supplementary Figure 2. Molecular phylogenetic analysis of amino acid sequences of orthologous genes for five of the six S genes of interest. The orthogroup for DND1 only included three genes (One *A. thaliana* susceptibility gene, one *T. aestivum* ortholog, and one *B. distachyon* ortholog) and so no phylogenetic tree was generated. Scale bars represent nucleotide substitutions per site.

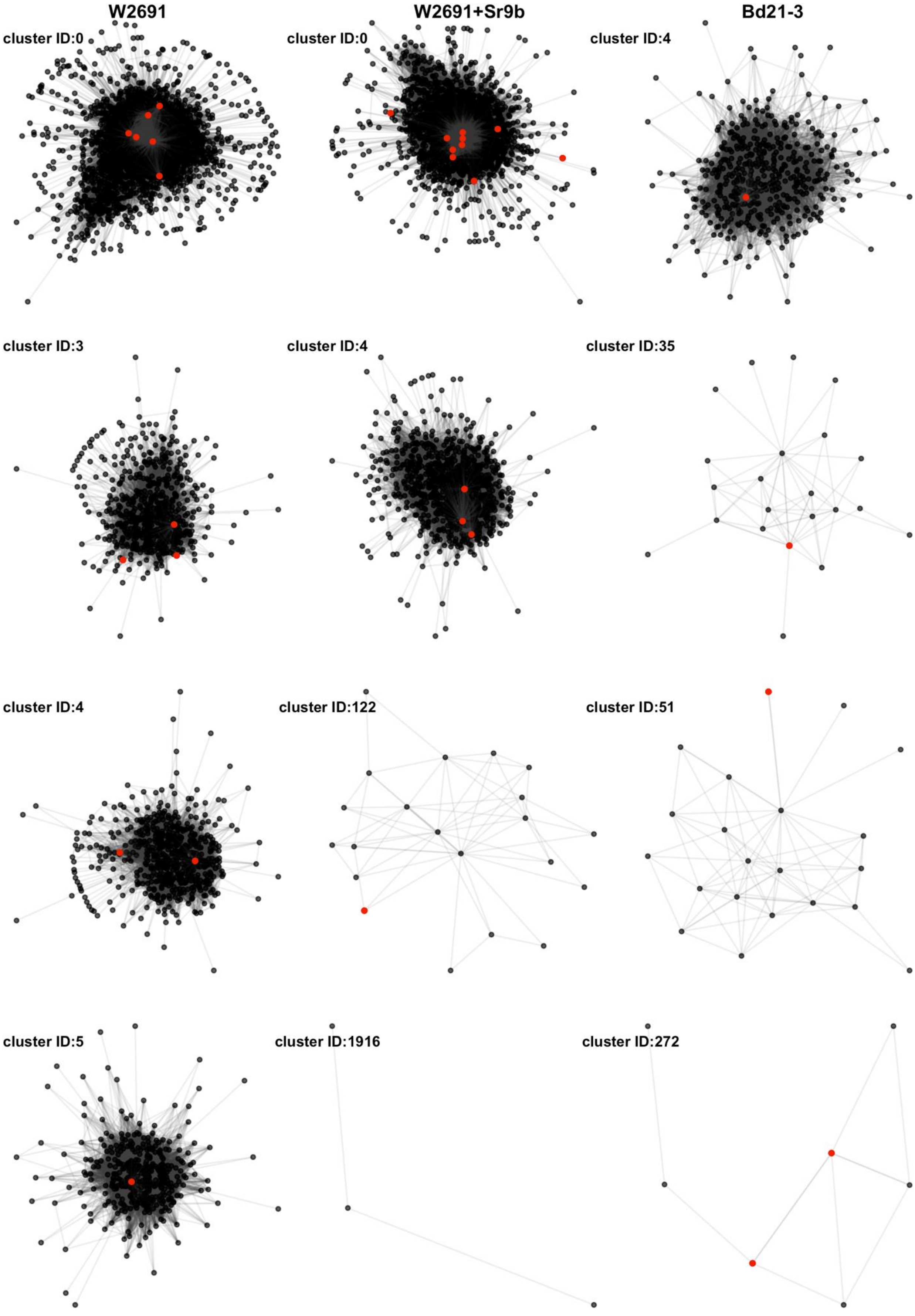
W2691
W2691+Sr9b
Bd21-3
cluster ID:0
cluster ID:0
cluster ID:4
cluster ID:3
cluster ID:4
cluster ID:35
cluster ID:4
cluster ID:122
cluster ID:51
cluster ID:5
cluster ID:1916
cluster ID:272

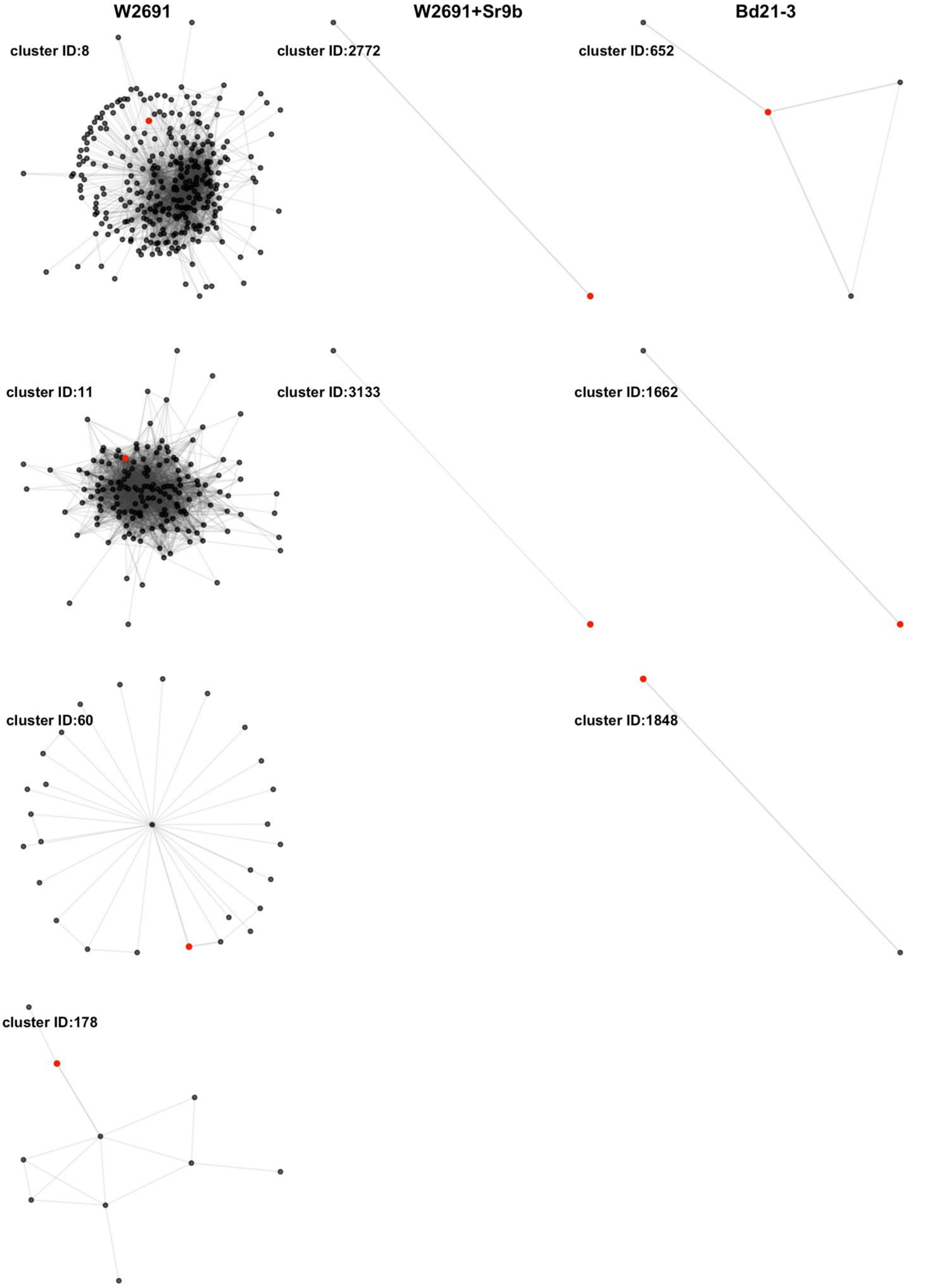
W2691
W2691+Sr9b
Bd21-3
cluster ID:8
cluster ID:2772
cluster ID:652
cluster ID:11
cluster ID:3133
cluster ID:1662
cluster ID:60
cluster ID:1848
cluster ID:178

Supplementary Figure 3. All clusters (nodes > 1) containing a *T. aestivum* or *B. distachyon* ortholog of the eight susceptibility candidates. Red points represent *T. aestivum* and *B. distachyon* orthologs of *S* genes. Gene IDs of members in co-expression clusters are presented in Table S9.

Table S1. RNA-seq reads, NCBI accession numbers and mapping statistics (excel file).

Table S2. Enriched GO terms (Observed count over expected count) among up- and down-regulated genes at three time points across the two *T. aestivum* genotypes and *B. distachyon* Bd21-3.

Table S3. List of candidate susceptibility genes and orthogroups (excel file).

Table S4. List of orthogroups containing two or more genes including gene IDs (excel file).

Table S5. List of singleton orthogroups and gene IDs (excel file).

Table S6. Average gene expression (FPKM), orthogroup, GO terms, and cluster numbers associated with all genes in W2691, W2691+*Sr9b*, Bd21-3 (excel file).

Table S7. Average gene expression (FPKM), orthogroup, GO terms, and cluster numbers associated with eight *S* gene candidates in W2691, W2691+*Sr9b*, Bd21-3 (excel file).

Table S8. Co-expression network data for W2691, W2691+*Sr9b*, Bd21-3 genotypes using the mock and infected RNA-seq data (text file, see github page https://github.com/henni164/stem_rust_susceptibility).

Table S9. Co-expression network data for clusters containing *S* gene candidates (text file, see github page https://github.com/henni164/stem_rust_susceptibility).
